# Damage analysis and optimal design of micro-structure milling cutter based on peridynamics

**DOI:** 10.1371/journal.pone.0307940

**Published:** 2024-12-10

**Authors:** Zhongwei Ren, Jing Deng, Hongwan Jiang, Sen Yuan, Xi Yue, Chuchun Tian

**Affiliations:** 1 Guizhou Institute of Technology, School of Mechanical Engineering, Guiyang, China; 2 School of Mechanical Engineering, Guizhou University, Guiyang, China; 3 Guizhou Key Laboratory of Special Equipment and Manufacturing Technology, Guizhou University, Guiyang, China; TU Dublin Blanchardstown Campus: Technological University Dublin - Blanchardstown Campus, IRELAND

## Abstract

H13 die steel has the characteristics of high hardness, strong toughness, and good heat resistance, and is a typical difficult to process materials material. During the cutting process, it is prone to accelerate tool wear and cause thermal deformation. By reasonably designing micro-grooves, the comprehensive performance of the tool can be effectively improved. In this study, by optimizing the structural parameters of the micro-groove, the comprehensive performance of the tool is significantly improved, and the micro-groove optimization control mechanism is deeply analyzed. At the same time, the micro-damage problem is numerically analyzed by using the peridynamics numerical simulation and comparison experiment. Research results indicate that properly increasing the distance between the slot at the outer contour of the cutting tool and the cutting edge and projecting it in a flattened shape onto the surface of the tool, ensures a smooth transition between the groove top and bottom near the cutting edge can effectively enhance the comprehensive performance of the cutting tool. The tool’s major cutting edge near-field and rake face is prone to micro-cracks resulting in crack diffusion. When the milling time is 3.5×10^−6^ s, the tool’s major cutting edge combined displacement increases most rapidly, the major flank optimization effect is the most obvious, and the resultant displacement is reduced by about 37.06%. By optimizing the structural parameters of micro-grooves on the rake face, this study enhances the comprehensive performance of the tool and unveils the formation, distribution, and variation patterns of near-field cracks on the tool’s cutting edge. The research results have certain valuable insights for the optimization design and manufacturing of high performance milling tools made from H13 die steel.

## Introduction

Difficult-to-machine materials are materials with excellent mechanical properties such as high specific strength and good wear resistance. They easily cause premature tool failure and reduce tool life in machining processes [1]. H13 steel, as a typical difficult-to-machine material, is subjected to high temperature, high pressure, large cutting force [2], and intermittent cutting [3] during milling. The surface of the tool is often accompanied by cracks, edge breakage, damage, and other phenomena [4], especially for the cutting edge which is mainly affected by thermal load and mechanical load, the damage resistance of the cutting edge decreases significantly. Therefore, it is particularly important to design a milling cutter with good damage resistance and long tool service life under impact load.

Researchers at home and abroad have found that micro-nano structures of certain shapes and sizes placed on the surface of the tool can effectively enhance the tool’s performance. Wu et al. [5] found that the surface temperature of the micro-structure tool was effectively reduced through comparative analysis of the front tool surface micro-structure tool and the conventional tool. According to the research of Xing et al. [6], they used nanosecond lasers to prepare microchannels on ceramic tools and evaluated their performance through turning experiments, finding that this method can effectively reduce cutting temperature and cutting force during cutting. Jiang et al. [7] carried out a study on carbide cutting tools by machining bionic groove structures with a certain size and distribution. Under the action of biomimetic microstructures, the efficiency of tool use has been effectively improved.

Given this, to effectively solve the above problems, improve the near-field damage resistance of the cutting edge tool, and extend the service life of the tool, many scholars at home and abroad are committed to optimizing the design of the micro-structure tool to improve the cutting performance of the tool. In the design of the micro-structure tool, the design of the micro-milling cutter needs to meet the stiffness, chip space, and other factors [8]. Du [9] used a simulation data-driven approach to design micro-slots on the rake face. Some scholars [10] improved the performance of the tool in green cutting by using biological structure as a reference for the bionic design of the tool microstructure. Some scholars also chose genetic algorithm to optimize the micro-structure tool [11,12], which improved the cutting performance of the micro-structure tool. However, there is little research on the microstructure optimization of the near-field damage resistance of cutting edge. According to previous studies [13], it is known that the tool is prone to brittle fracture when subjected to impact load. The main reason for brittle fracture is caused by the formation of microcracks. These microcracks form when, under alternating loads, the weak links inside the material are subjected to forces that exceed their ultimate resistance. With the continuous application of load, these micro-cracks gradually expand and develop into obvious macroscopic cracks [14]. Under the action of heat-mechanical load, the failure mechanism of the tool goes through the progressive process of damage accumulation → crack initiation → expansion → tool failure [15]. Therefore, when optimizing the microstructure of the tool, it is very important to consider factors such as temperature and impact load as indicators, which helps to efficiently improve the damage resistance of the tool and enhance the damage resistance of the tool.

The optimal design of tool structure is often carried out by the finite element method. However, in the analysis of damage, cracks, etc., the analysis results often have a strong dependence on the grid division [16], and it is difficult to deal with the problems of cross and connectivity between multiple cracks [17]. The emergence of peridynamics (PD) can solve such problems well. The PD theory was first proposed by Silling in 2000 [18], which is a method of uniform discretization of matter into a series of material points with physical information, and the meshless model is realized by using bonds between material points. It has the advantages of molecular dynamics, no grid, and no singularity, so it has a great application prospect in the field of crack generation and propagation. Xu et al. [19] simulated damage in the indentation and scratching of 3C-SiC and studied the generation of free radical cracks on both sides of the groove. The experimental results confirmed that PD theory could be used to describe the scratch damage of brittle materials. Liu et al. [20] established a dynamic model of the impact of a split Hopkinson bar on a single crack circular orifice plate with PD theory and calculated the crack initiation toughness. Zhang et al. [21] adopted the stochastic PD method to simulate the crack growth process of rocks with defects. Ren et al. [22] used PD theory to predict the brittle damage near-field the edge of a cemented carbide micro-slot car. It is found that the simulation results have a controllable low error rate. Liu et al. [23] established a conventional state PD numerical model suitable for Ti2AlNb cutting simulation. The results show that the PD simulation can accurately simulate the deformation and damage evolution of Ti2AlNb cutting chips. However, there are few reports on PD research of tools with complex geometric profiles, especially complex surface microstructures.

Based on the research status, this study proposes a method that combines finite element analysis with mesh-free techniques. Based on the peridynamics theory, it conducts research on the optimization and regulation mechanism of complex surface microstructure tools, as well as the formation, distribution, and variation trends of near-field cracks on cutting edges. Cleverly implemented numerical analysis of the near-field crack evolution process on complex surface cutting edges of cutting tools based on the PD theory. Established a correlation model between tool microstructure and damage degree, as well as a mapping model between tool mechanical load and PD damage.

## Micro-structure design and PD theory fundamentals

### Design of micro-structure near-field the cutting edge

**Fig 1:**
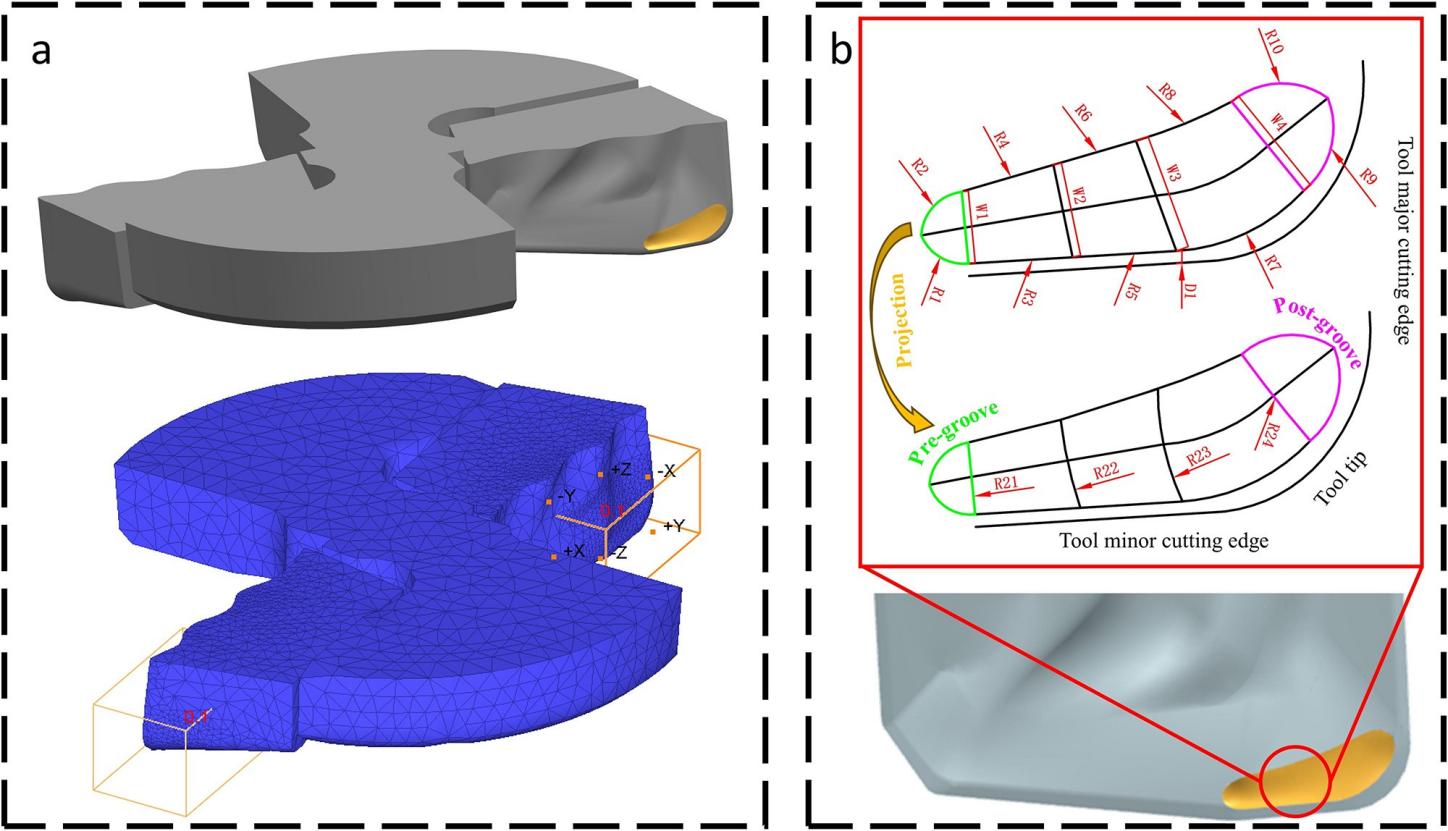
Cutting tool. (a) Deform model and meshing (b) Tool solid model and microgroove microstructure.

The main task of this paper is to verify the damage resistance of the tool under impact load. However, in the milling experiment, it is difficult to accurately obtain the duration of impact load, and it is difficult to accurately control the size and duration of impact load, so it is impossible to verify the difference of simulation through experiments. Therefore, this paper adopts the method of combining NX 3D modeling and deform finite element analysis software, saves the solid model as STL format through NX software, and imports it into DEFORM-2D /3D pre-processing, to build the finite element milling model under ideal conditions. The damage resistance of the tool under the impact load under ideal conditions is studied. In the pre-processing process, the mesh division plays a direct decisive role in the accuracy and reliability of the solution of the finite element analysis. The mesh accuracy of hexahedral mesh is theoretically higher than tetrahedral mesh, but when the geometry is complex, hexahedral mesh is difficult. Tetrahedral mesh is suitable for arbitrary geometry, remeshing is fast, and relatively accurate calculation results can be obtained. The tool studied in this paper has a complex surface structure, and the model needs to be meshing quickly in the simulation process. So, in both calculation accuracy and efficiency, and to ensure the reliability of the simulation, tetrahedral element mesh suitable for arbitrary geometry and easy to repartition are selected in this paper, and absolute mesh is used to partition the workpiece, the minimum size is 0.1mm, and the size ratio is 6. For the tool, relative meshing is selected, with the number of meshes being 60,000 and the size ratio also being 6. At the same time, for the milling cutter groove type, we carried out the local thinning treatment with a thinning ratio of 0.1. Fig 1 shows the schematic diagram of the tool structure and grid division.

Based on a comprehensive team effort and a large-scale simulation experiment, the key parameters of the tool depicted in Fig 1c were subjected to rigorous analysis to identify the ten most influential factors on the comprehensive performance of the tool. Under the milling speed of 150 m/min, cutting depth of 0.3mm, feed rate of 0.8mm /r, and total feed of 8mm, the comprehensive evaluation method was used to carry out single factor experiments with the key structural parameters of the tool as variables, and the four key parameters with the most significant influence were determined. For a comprehensive evaluation, the experiment selected the maximum surface temperature (MT), average temperature (AT), and maximum interface pressure (MIP) of the cutting tool during milling work, the weights of which were 0.45:0.45:0.1. Then through the orthogonal experiment, the optimal scheme of the tool is further determined. Table 1 shows in detail the single factor milling experiment scheme with 10 key parameters selected.

**Table 1:** Single factor milling experiment. Unit: mm.

Group	No.	R1	R7	R9	R22	R23	R24	W2	W3	W4	D1
**Ⅰ**	1	0.224	0.907	0.412	0.579	1.675	2.610	0.456	0.579	0.563	0.061
	2	0.235	0.907	0.412	0.579	1.675	2.610	0.456	0.579	0.563	0.061
	3	0.246	0.907	0.412	0.579	1.675	2.610	0.456	0.579	0.563	0.061
**Ⅱ**	1	0.224	0.907	0.412	0.579	1.675	2.610	0.456	0.579	0.563	0.061
	2	0.224	0.927	0.412	0.579	1.675	2.610	0.456	0.579	0.563	0.061
	3	0.224	0.947	0.412	0.579	1.675	2.610	0.456	0.579	0.563	0.061
**Ⅲ**	1	0.224	0.907	0.412	0.579	1.675	2.610	0.456	0.579	0.563	0.061
	2	0.224	0.907	0.612	0.579	1.675	2.610	0.456	0.579	0.563	0.061
	3	0.224	0.907	0.712	0.579	1.675	2.610	0.456	0.579	0.563	0.061
**Ⅳ**	1	0.224	0.907	0.412	0.579	1.675	2.610	0.456	0.579	0.563	0.061
	2	0.224	0.907	0.412	0.619	1.675	2.610	0.456	0.579	0.563	0.061
	3	0.224	0.907	0.412	0.659	1.675	2.610	0.456	0.579	0.563	0.061
**Ⅴ**	1	0.224	0.907	0.412	0.579	1.675	2.610	0.456	0.579	0.563	0.061
	2	0.224	0.907	0.412	0.579	3.675	2.610	0.456	0.579	0.563	0.061
	3	0.224	0.907	0.412	0.579	5.675	2.610	0.456	0.579	0.563	0.061
**Ⅵ**	1	0.224	0.907	0.412	0.579	1.675	2.610	0.456	0.579	0.563	0.061
	2	0.224	0.907	0.412	0.579	1.675	1.610	0.456	0.579	0.563	0.061
	3	0.224	0.907	0.412	0.579	1.675	3.310	0.456	0.579	0.563	0.061
**Ⅶ**	1	0.224	0.907	0.412	0.579	1.675	2.610	0.456	0.579	0.563	0.061
	2	0.224	0.907	0.412	0.579	1.675	2.610	0.466	0.579	0.563	0.061
	3	0.224	0.907	0.412	0.579	1.675	2.610	0.476	0.579	0.563	0.061
**Ⅷ**	1	0.224	0.907	0.412	0.579	1.675	2.610	0.456	0.579	0.563	0.061
	2	0.224	0.907	0.412	0.579	1.675	2.610	0.456	0.584	0.563	0.061
	3	0.224	0.907	0.412	0.579	1.675	2.610	0.456	0.589	0.563	0.061
**Ⅸ**	1	0.224	0.907	0.412	0.579	1.675	2.610	0.456	0.579	0.563	0.061
	2	0.224	0.907	0.412	0.579	1.675	2.610	0.456	0.579	0.543	0.061
	3	0.224	0.907	0.412	0.579	1.675	2.610	0.456	0.579	0.553	0.061
**Ⅹ**	1	0.224	0.907	0.412	0.579	1.675	2.610	0.456	0.579	0.563	0.061
	2	0.224	0.907	0.412	0.579	1.675	2.610	0.456	0.579	0.563	0.071
	3	0.224	0.907	0.412	0.579	1.675	2.610	0.456	0.579	0.563	0.081

In the Deform milling simulation, to ensure the good convergence of the calculation results, the conjugate gradient method solver is selected in this paper, and the direct iteration method is used to calculate. At the same time, to reduce the distortion problem in the calculation, the Lagrange solution type [24] is selected to better repartition the grid. In terms of tool wear type, the Usui model, which performs better in milling, is adopted, and correction factors a = 0.00001 and b = 1000 are set according to experience. Milling simulation parameters are set in Table 2.

**Table 2:** Set the main parameters of the deform simulation.

Environment Temperature(°C)	Cutting tool	Friction coefficient	Heat transfer coefficient (N/sec/mm/C)	Convection coefficient (N/sec/mm/C)
20	WC	0.4	1200	10
Line speed (mm/sec)	Workpiece	Number of simulation steps	Sec/Step	Rotate speed (rad/s)
39.81	AISI-H-13	5000	4.0192e^-5^	312.64

### PD theory

**Fig 2:**
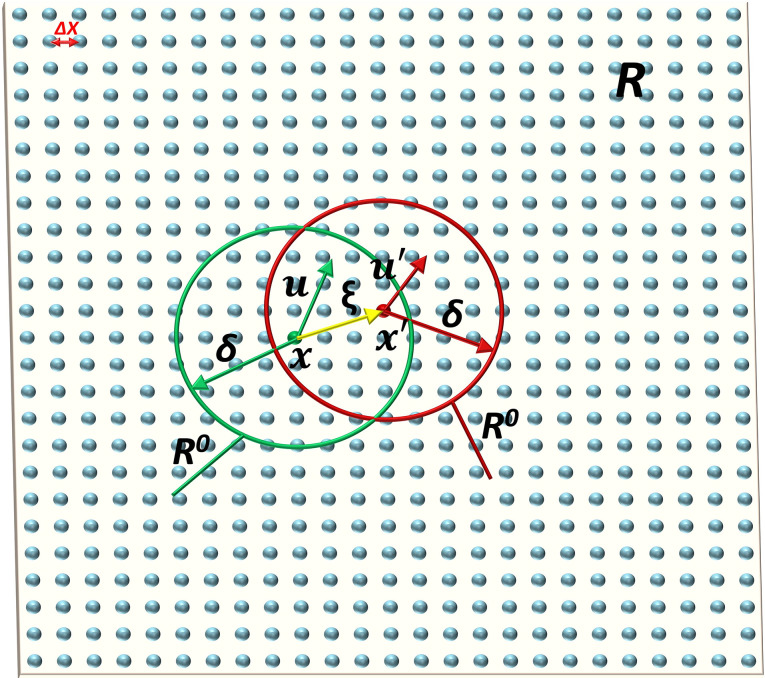
The interaction force between material points.

The PD theory assumes that an object is located within a hypothetical spatial domain *R*. The material points within a subdomain *R*^*0*^, with a radius equal to the near-field range *δ*, interact with each other through the force ***f*** as shown in Fig 2. By utilizing Newton’s second law, the "constitutive force function" equation at time t for the material points can be determined [25].


$$\rho (x)\overset{¨}{u}(x,t)=\int_{R^{0}}f(x,x^{'},u(x,t),u(x^{'},t),t)dV_{x'}+b(x,t)$$ (1)

Where, ***u*** and ***u***’ respectively represent the displacement of material points ***x*** and *x*’ at time t; *ρ* is the material density; *b*(*x*,*t*) is external body force; *dV*_*x*’_ is the volume of material point ***x***’.


$$R^{0}=R^{0}(x^{'},\delta )=\{x^{'}\in R,\|x^{'}-x\|\leq \delta \}$$ (2)

Let *L*_*u*_(***x***,*t*) be the internal force caused by the interaction between ***x*** and *x*’ in the near-field range in the space domain *R*, then:

$$L_{u}(x,t)=\int_{R^{0}}f(x,x^{'},u(x,t),u(x^{'},t),t)dV_{x'}$$ (3)

**Fig 3:**
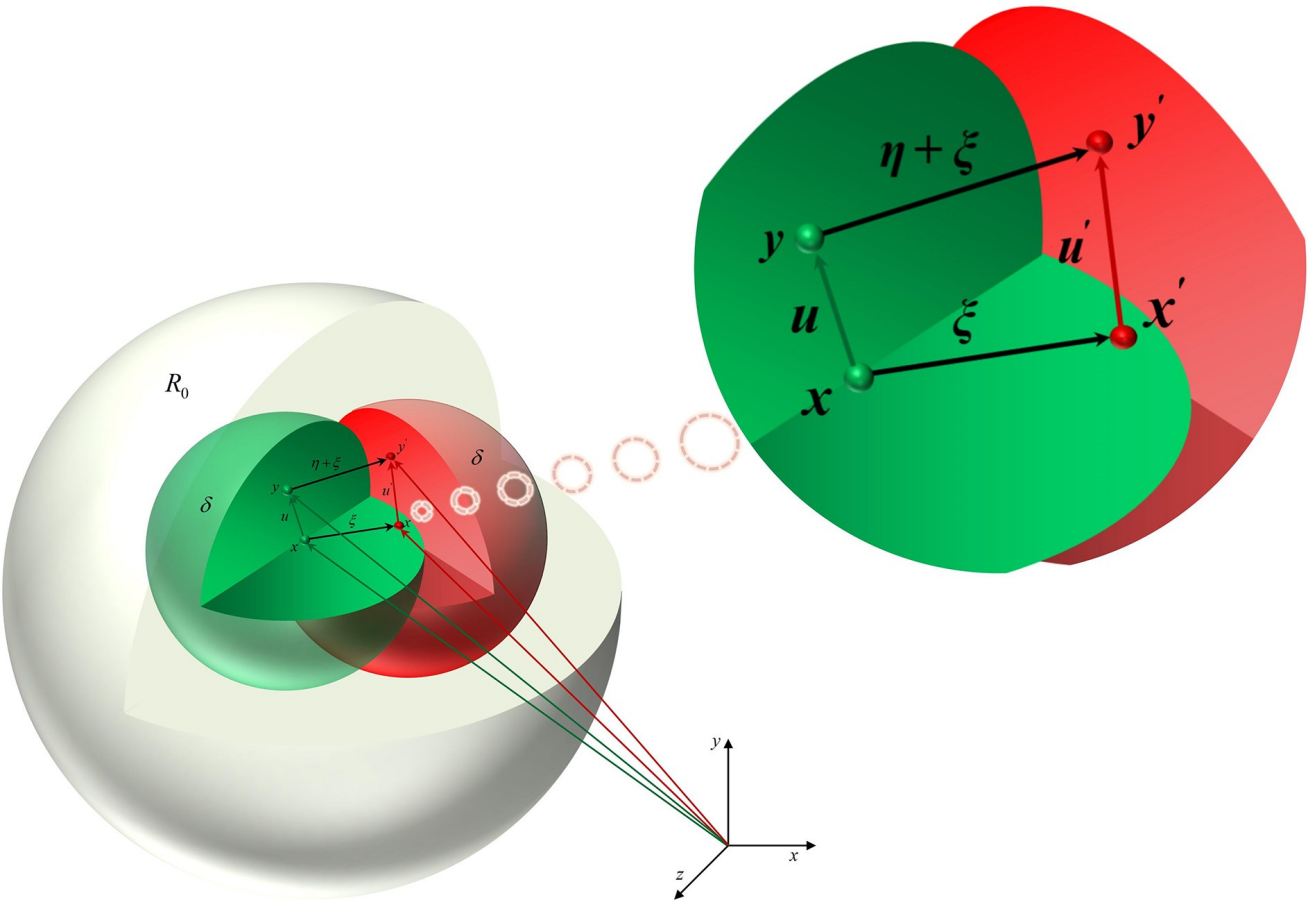
The relationship of motion states between material points.

At this point, introducing two physical quantities *ξ* and ***η***, the *ξ* = ***x***’−*x* is defined as the relative position of material points ***x*** with respect to *x*’ before deformation, and ***η*** = ***u***’−***u*** as the relative displacement of material points *x* with respect to *x*’ after time *t* [26]. The motion state between material points is illustrated in Fig 3, and Eqs (1) and (3) yield the following translation:

$$\rho (x)\overset{¨}{u}(x,t)=L_{u}(x)+b(x,t)=\int_{R^{0}}f(\eta ,\xi )dV_{x'}+b(x,t)$$ (4)

According to Newton’s third law:

f(−η,−ξ)=−f(η,ξ) (5)

When two material points move relative, the interaction force ***f*** between the two material points is consistent with the direction of the relative position and the relative displacement vector *η*+*ξ*, then:

f(η,ξ)×(η+ξ)=0 (6)

According to Formula (5) and Formula (6):

f(η,ξ)=F(η,ξ)⋅(η+ξ)∀η,ξ (7)

When the research object is a microelastic material, its "constitutive force function" is expressed as:

$$f(\eta ,\xi )=\frac{\eta +\xi }{\left|\eta +\xi \right|}\cdot c\cdot s(\eta ,\xi )\cdot \mu (\xi ,t)$$ (8)

Where *c* is the material bond constant, and its value is related to bulk modulus *K* and near-field range *δ*. *c* is defined as follows in three-dimensional space [27]:

$$c=\frac{18K}{\pi \delta^{4}}$$ (9)

*s*(***η***,*ξ*) is called the elongation of the bond between material points [28].


$$s=\frac{\left|\eta +\xi |-|\xi \right|}{\left|\xi \right|}$$ (10)

Use *μ*(*ξ*,*t*) to indicate the damage between material points.


$$\mu (\xi ,t)=\{\begin{array}{ll} 1 & s<s_{0}\\ 0 & \mathrm{else} \end{array}$$ (11)

Where *s*_0_ is the ultimate elongation of the bond between two material points. When the elongation of the "bond" between two material points is greater than *s*_0_, the bond between the point pair will break. The size is related to the critical energy release rate of the material *G*_0_ [29], which is expressed as follows:

$$s_{0}=\sqrt{\frac{5G_{0}}{9K\delta }}$$ (12)

When there is no interaction force between the two material point bonds, the damage at point A can be defined as:

$$\varphi (x,t)=1-\frac{\int_{R^{0}}\mu (\xi ,t)dV_{x'}}{\int_{R^{0}}dV_{x'}}$$ (13)

*φ*(***x***,*t*) represents the degree of damage of material point *x* at time t. 0≤*φ*≤1, When *φ* = 1, it means that the "bond" at the material point *x* is completely broken.

According to the basic theory of PD, the interaction between two material points can be established through bonds in the near-field range with a radius of *δ*. However, when constructing PD models, only the case where the central node coordinate is in the near-field range *δ* is usually considered, while ignoring the local area where the node coordinate is located outside the near-field range *δ*. For this purpose, a volume reduction factor *β* [30] was introduced to reduce errors. Which is expressed as follows:

$$\beta =\{\begin{array}{l} \;1\;|\xi |\leq \delta -0.5\Delta x\\ \frac{\delta +0.5\Delta x-|\xi |}{\Delta x}\;\delta -0.5\Delta x<|\xi |\leq \delta +0.5\Delta x\\ \;0\;esle \end{array}$$ (14)

After introducing the volume reduction factor *β*, the ’constitutive force function’ can be expressed as:

$$\rho (x)\overset{¨}{u}(x,t)=\int_{R^{0}}\frac{\eta +\xi }{\left|\eta +\xi \right|}\cdot c\cdot s(\eta ,\xi )\cdot \mu (\xi ,t)\cdot \beta \cdot dV_{x'}+b(x,t)$$ (15)

### Construction and calculation of PD model of complex surface

**Fig 4:**
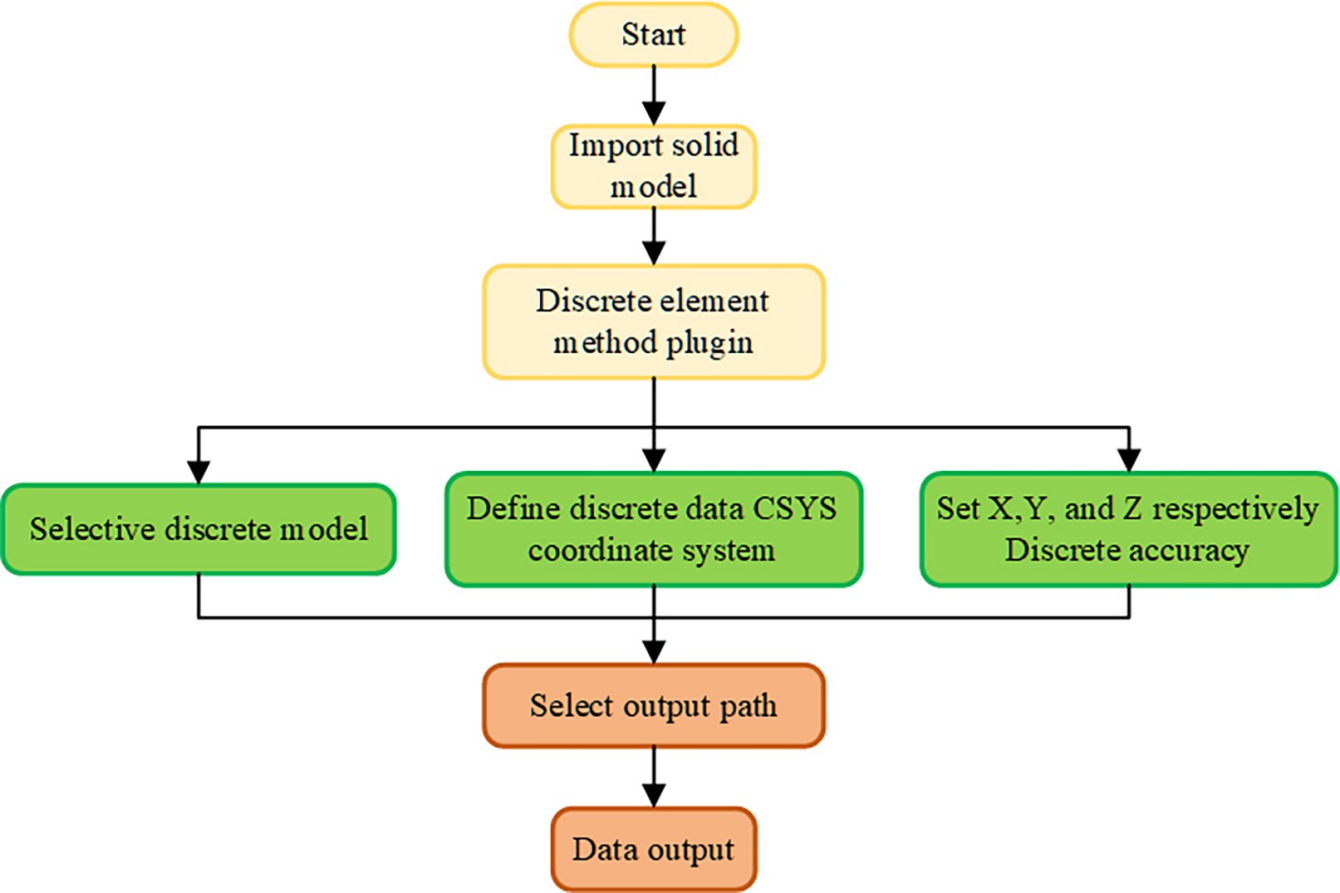
Discrete flow chart of complex surfaces.

The microstructure of complex curved micro-structured tools is largely determined by their surface morphology and structural features, which are often very small and complex. Using traditional methods for discretization may cause errors in boundary features and affect computational accuracy. To effectively solve this problem, this article has developed a PD dispersion plugin based on NX software, which is suitable for complex geometric solid models. It has achieved PD discretization processing for tools with complex surfaces. As the main task is to verify the tool’s resistance to damage under impact loads, the development process of the plugin is not overly detailed. Based on the five optimal schemes selected through orthogonal experiments, perform Deform simulation, using the maximum impact load in the simulation as the magnitude of the external force on the milling cutter in PD analysis, and study the damage situation of the milling cutter under extreme working conditions. Milling cutter density *ρ* = 14.5 g/cm^3^, bulk modulus K = 550 GPa, length 11 mm, width 7 mm, height 3.5 mm, net width 6.25 mm. Through multiple numerical simulations and analysis, taking into account the geometric dimensions and computational efficiency of the milling cutter, in this paper, the spatial unit volume Δ*x* = Δ*y* =Δ*z* = 0.07 mm, the unit volume of space V_*x*’_ = 3.43×10^−4^ mm, uniformly dispersed as 420,260 material points, the radius of the near-field range of material point is 3Δ*x* [31], the time step is *dt* = 1×10^−8^ s: The discrete flow of complex surfaces is shown in Fig 4.

**Fig 5:**
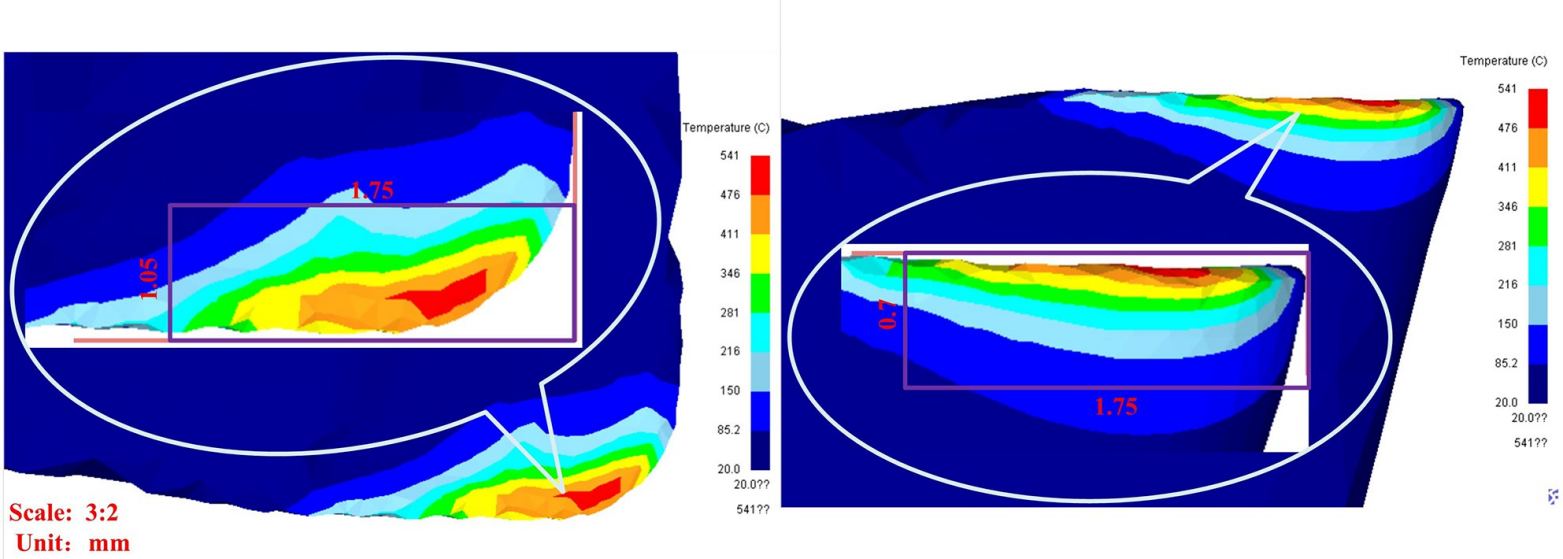
Impact load bearing area of milling cutter.

In PD numerical analysis, how to determine the impact load acting area during the milling process of the milling cutter is a key issue. In this study, milling conditions involve large feed rates and cutting depths, and the workpiece is made of H13 mold steel, which is a difficult to machine material, with high hardness and low deformation coefficient. Therefore, the impact load zone can be considered as a surface contact area with specific dimensions. By combining the simulation results of Deform with the structural features of the tool itself, the region can be simplified into a rectangular shape of a certain size, as shown in Fig 5.

**Fig 6:**
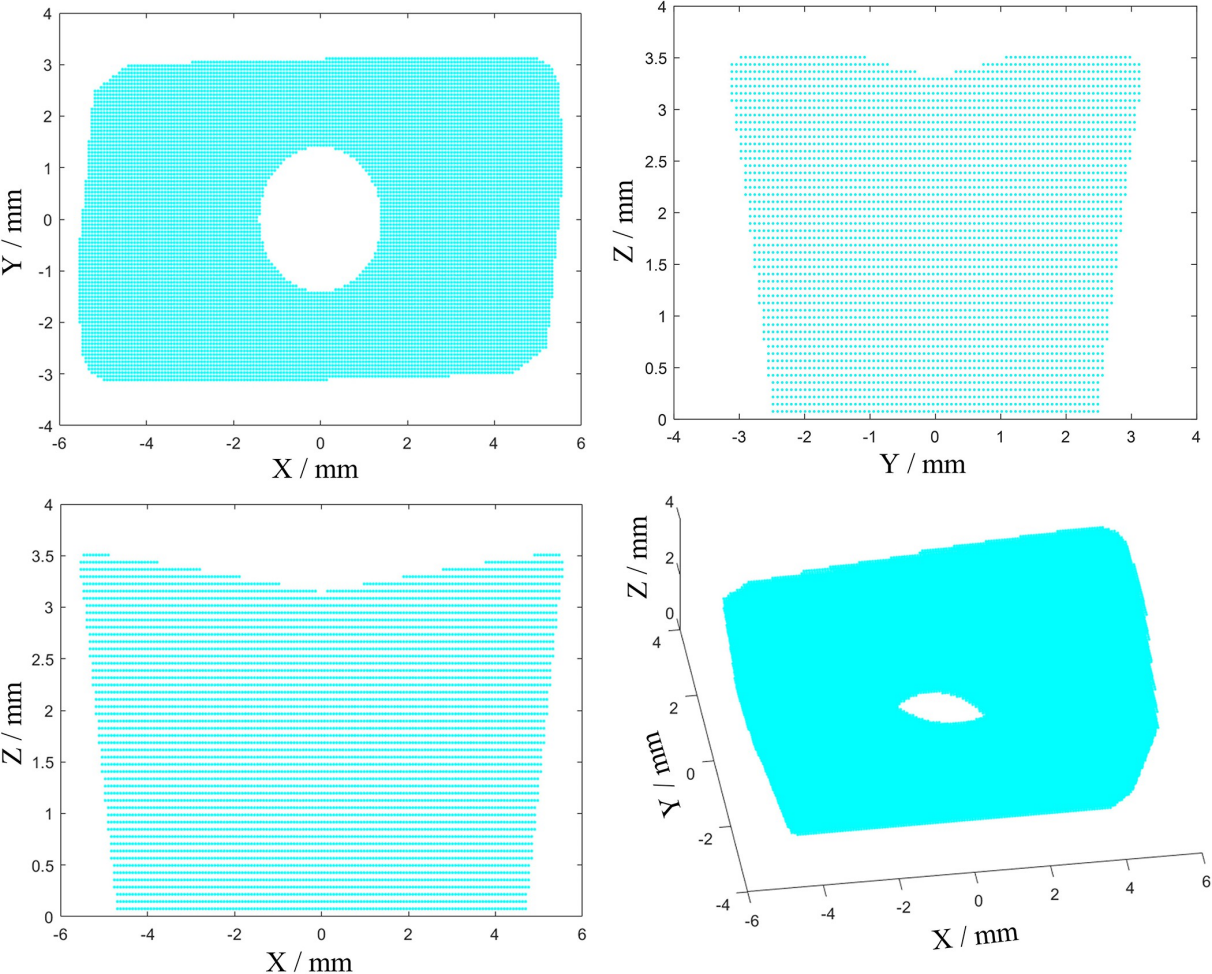
Discrete model of original milling cutter.

The milling cutter was discretized through plugin development, and the resulting discrete model of the milling cutter was visualized using scatter visualization in MATLAB, as shown in Fig 6.

The number of material points in the force area of the cutter is defined as *P*, and the maximum impact load collected in the simulation experiment is considered as *F*, which is used as the external load received in PD numerical analysis. This external load uniformly acts on all material points in the force area. On the contrary, the external load on the material points outside the stress area is zero. When t = 0, the initial force situation of each material point in the stress area is:

$$f_{i}=\frac{F}{P}$$ (16)

According to Eq 1, the initial acceleration of a material point in the stress area can be calculated as:

$$a_{i}=\frac{f_{i}}{\rho \Delta x^{3}}$$ (17)

The initial velocity and displacement of the corresponding material point are expressed as:

$$v_{i}=a_{i}\Delta t$$ (18)

$$s_{i}=\frac{1}{2}a_{i}\Delta t^{2}$$ (19)

**Fig 7:**
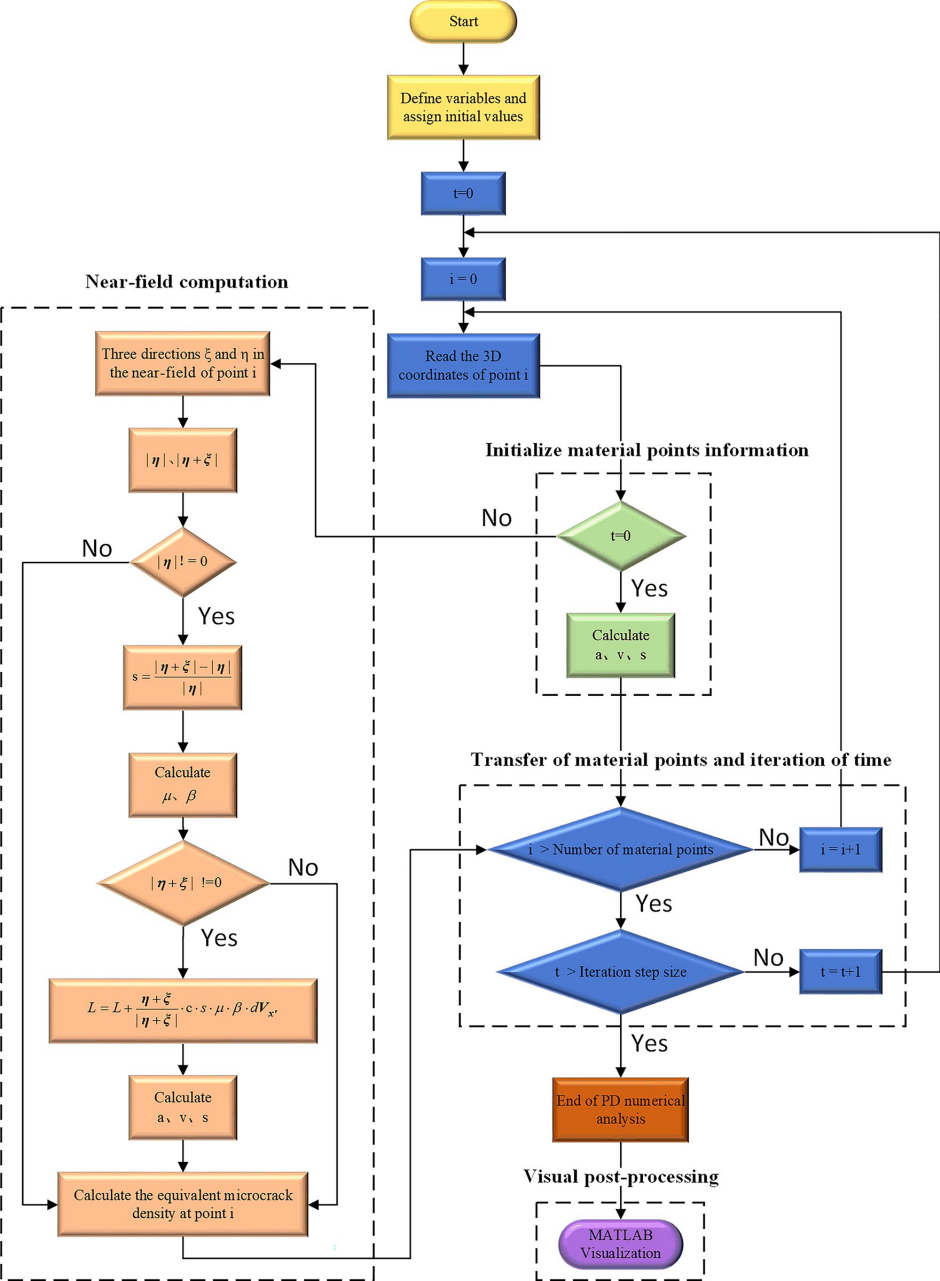
PD numerical analysis process.

Perform cyclic iteration with the initial acceleration as the initial state to obtain the motion of all material points of the milling cutter. Control the number of iterations by defining a time step Δ*t*, and output the displacement three-dimensional coordinates of each material point under the defined time step. The PD numerical analysis program flow is shown in Fig 7.

## Results

### Simulation experiment results

**Fig 8:**
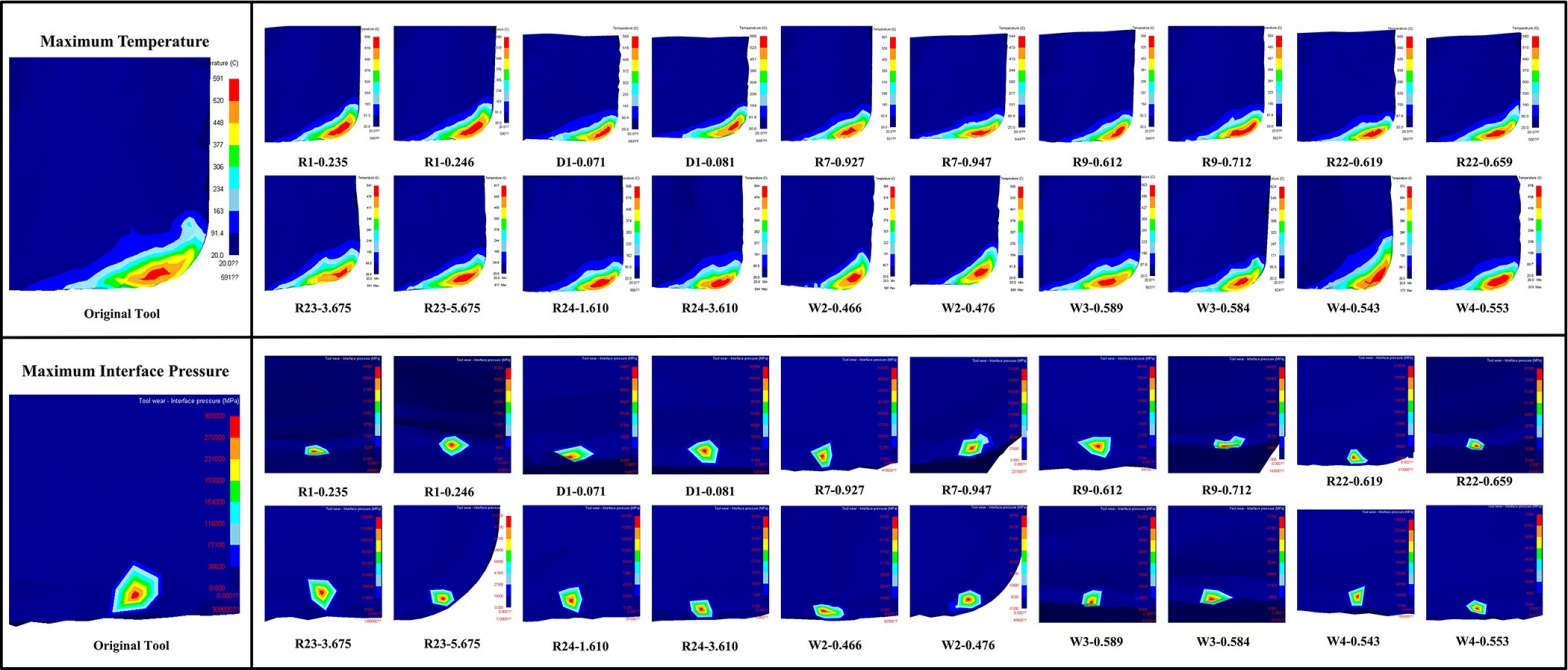
Simulation nephograms.

To improve simulation efficiency, we specifically studied the evaluation criteria when the milling simulation is set to half of the predetermined working time. Fig 8 shows the distribution of the MP and MIP on the tool surface with the change in the tool microgroove structure.

**Fig 9:**
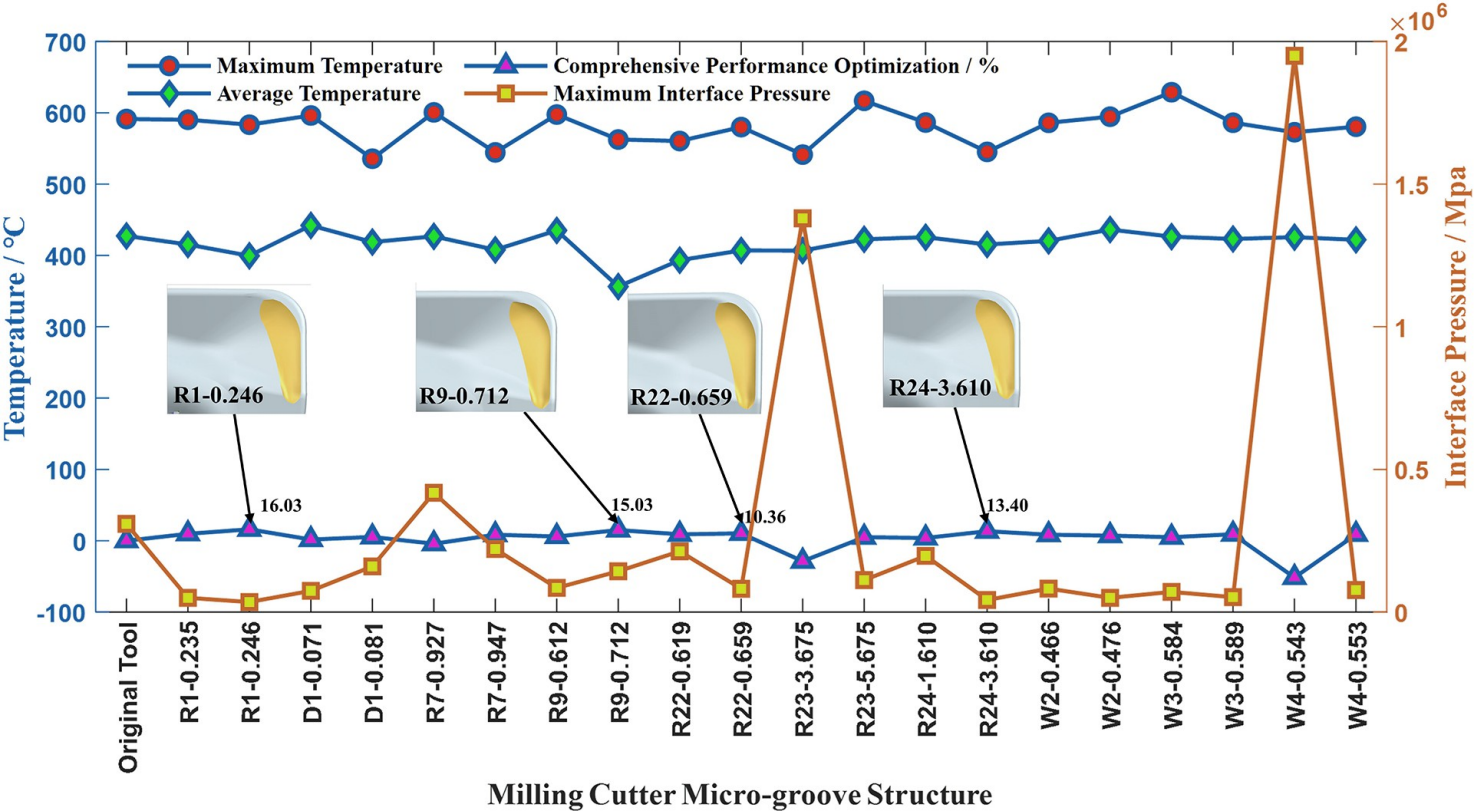
Milling simulation results.

The relationship between three evaluation indicators and micro-groove curvature can be observed in Fig 9. It is evident from the graph that curvatures R1, R9, R22, and R24 exhibit the most significant impact on enhancing the comprehensive performance of the cutting tool. As the key micro-groove curvatures vary, the increase in tool surface temperature can lead to either an increase or decrease in MIP. This indicates that temperature and MIP indicators do not exhibit a monotonic change to a specific parameter variation. The change trend of only the pre-groove curvature R1 and the post-groove depth curvature R24 is consistent among the three evaluation indicators for curvature. That is, the three indicators decrease monotonically with the increase of R1, while they show an increasing trend followed by a decrease with R24. When the curvature R1 increases, the distance between the pre-groove and the tool’s minor cutting edge increases, resulting in a flattened projection on the tool surface, leading to a decrease in all three indicators, indicating better comprehensive performance of the tool. Increasing or decreasing the post-groove depth curvature R24 can enhance the comprehensive performance of the cutting tool. However, when the curvature R24 is increased, a relatively notable transitional characteristic is observed between the top and bottom of the pre-groove near and close to the tool’s major cutting edge. At this point, the reduction in all three indicators is greater than the decrease in curvature R24, resulting in a higher enhancement in the comprehensive performance of the cutting tool than when reducing the R24 curvature radius. Increasing the post-groove curvature R9 results in an increased distance between the post-groove and the tool’s major cutting edge, causing a flattened projection on the tool surface. This reduces the MIP of the tool but leads to an increase in surface temperature, resulting in a lower improvement in the comprehensive performance of the tool. However, when the curvature R9 continues to increase to a certain extent, although the MIP indicator slightly increases, the surface temperature is effectively reduced. This leads to a more significant improvement in the comprehensive performance of the tool. Increasing the depth curvature of the pre-groove R22, located in the pre-groove area near the cutting edge and the tool minor cutting edge, the distance between the groove top and the bottom of the groove presents a transition region, which will make the MT and AT of the tool surface first decrease and then increase, but still lower than the temperature without optimization, and the MIP is continuously reduced, and the comprehensive performance of the tool continues to increase.

In summary, by adjusting the curvatures R1 and R9, the distance between the pre-groove and post-groove of the microgroove and the minor cutting edge and the major cutting edge can be effectively changed. Adjusting the curvatures R22 and R24 can produce a smooth transition effect between the top and bottom of the microgroove grooves close to the cutting edge, thereby significantly improving the tool performance.

Considering the aforementioned analysis, the distance between the pre-groove and post-groove of the microgroove and the cutting edge, as well as the reasonable transition effect between the top and bottom of the microgroove groove close to the cutting edge, are the key factors affecting the performance of the tool. So, orthogonal experiments were conducted on the curvature radii R1, R9, R22, and R24, focusing on maximizing tool performance through the highest optimization amplitude, while disregarding the potential impact of interaction [32]. The orthogonal table L9(3^4^) was selected for the experiments. The simulation results of orthogonal milling are presented in Table 3.

**Table 3:** Orthogonal experimental results.

No.	Structural parameters				Simulation result		
	R1/mm	R9/mm	R22/mm	R24/mm	MT/°C	AT/°C	MIP / MPa
**1**	0.240	0.612	0.659	3.61	610.00	421.02	2.29E+05
**2**	0.240	0.812	0.739	4.61	579.33	440.68	2.25E+05
**3**	0.240	1.012	0.819	5.61	620.59	449.64	9.27E+04
**4**	0.256	0.612	0.659	4.61	614.17	444.92	5.76E+05
**5**	0.256	0.812	0.739	5.61	582.85	416.19	1.03E+05
**6**	0.256	1.012	0.819	3.61	674.9	473.62	2.33E+05
**7**	0.272	0.612	0.739	3.61	549.24	409.25	7.28E+05
**8**	0.272	0.812	0.819	4.61	561.58	412.63	2.79E+05
**9**	0.272	1.012	0.659	5.61	565.77	424.97	1.84E+05

The three evaluation indexes in this paper are of different importance, and the membership degree of each experimental index is transformed according to the weight ratio [33]. The optimization of the maximum surface temperature, average temperature, and maximum interface pressure is represented by MTO, ATO, and MIPO respectively, and the membership degree is represented by MPM, ATM, and MIPM respectively, and the simulation comprehensive score is calculated. The calculation results are shown in Table 4.

**Table 4:** Comprehensive score.

No.	MTO	ATO	MIPO	MPM	ATM	MIPM	Comprehensive score
**1**	4.30%	4.11%	25.89%	51.65%	81.72%	78.55%	67.87%
**2**	9.11%	-0.37%	27.18%	76.05%	51.17%	79.18%	65.17%
**3**	2.64%	-2.41%	70.00%	43.22%	37.25%	100.00%	46.21%
**4**	3.64%	-1.33%	-86.41%	48.33%	44.59%	23.93%	44.20%
**5**	8.56%	5.21%	66.67%	73.25%	89.22%	98.38%	82.95%
**6**	-5.88%	-7.87%	24.60%	0.00%	0.00%	77.92%	7.79%
**7**	13.83%	6.79%	-135.60%	100.00%	100.00%	0.00%	90.00%
**8**	11.90%	6.02%	9.71%	90.18%	94.75%	70.68%	90.29%
**9**	11.24%	3.21%	40.45%	86.85%	75.58%	85.63%	81.65%

The range analysis of the comprehensive score is carried out, and the calculation results are shown in Table 5.

**Table 5:** Range analysis.

Factors Project	A-R1(mm)	B-R9(mm)	C-R22(mm)	D-R24(mm)
**K1**	179.25%	202.07%	193.73%	165.66%
**K2**	134.95%	238.41%	238.12%	199.66%
**K3**	261.94%	135.66%	144.29%	210.82%
**Range**	126.99%	102.75%	93.83%	45.16%
**Primary and** **secondary factors**	ABCD			
**Optimal solution**	A_3_B_2_C_2_D_3_			

**Fig 10:**
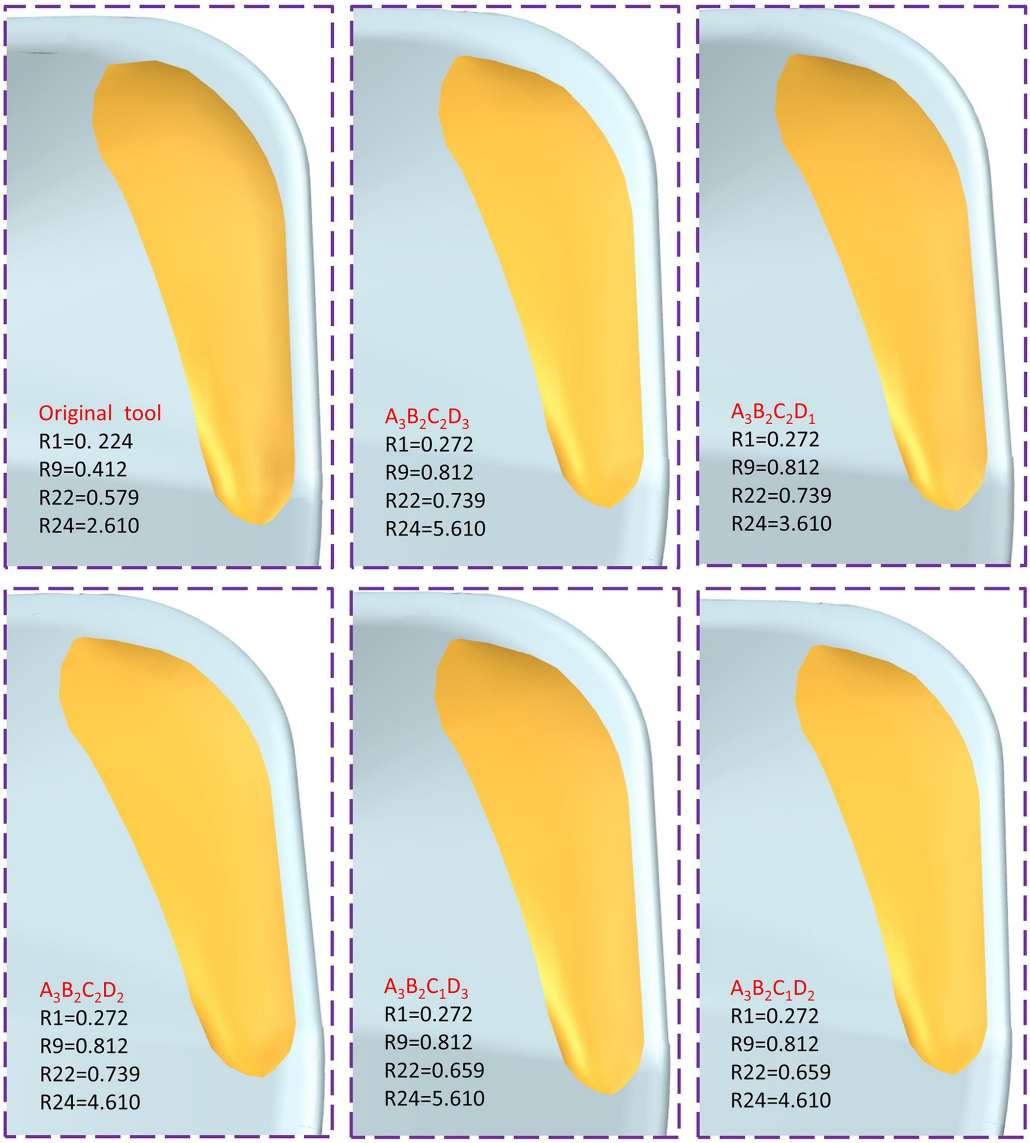
Optimal solution for milling cutter.

From the range analysis, it can be seen that the order of influence on the comprehensive performance of the tool is R1>R9>R22>R24, and the optimal scheme is (R1)_3_(R9)_2_(R22)_2_(R24)_3_, that is, the radius of curvature of the inner circle of the pre-groove R1 = 0.272mm, and the radius of curvature of the inner circle of the post-groove R9 = 0.812mm. When the depth curvature radius of the slot is R22 = 0.739mm and R24 = 5.61mm, the scheme is optimal. According to the range analysis results, the optimal scheme was selected in the primary and secondary order of the influencing factors. The closer to the optimal scheme, the better the overall tool performance, so the optimal scheme was selected as A3B2C2D3> A3B2C2D1> A3B2C2D2> A3B2C1D2> A3B2C1D3, as shown in Fig 10.

### Numerical analysis and visualization results of PD damage cracks

**Fig 11:**
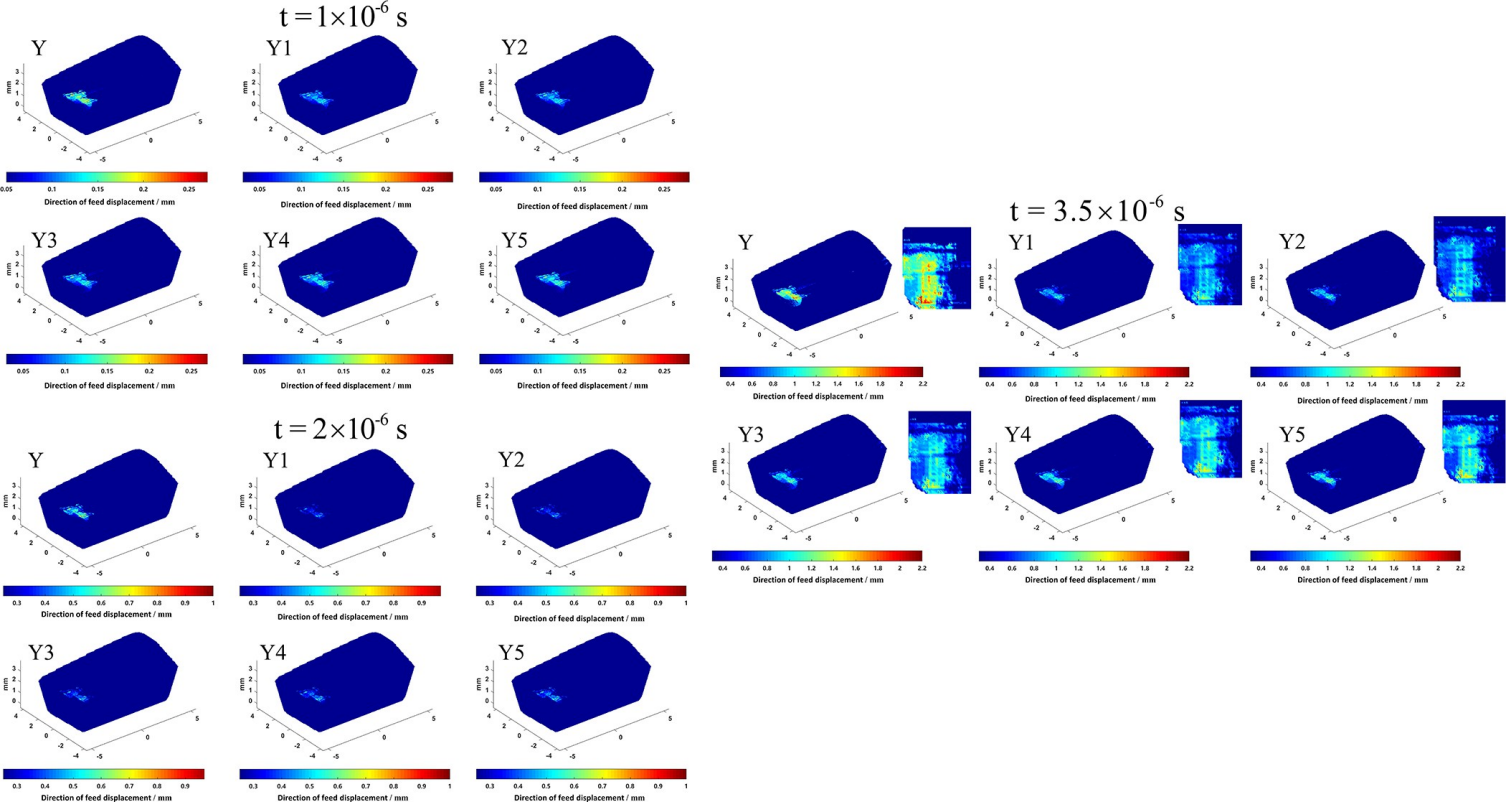
Tool direction of feed displacement changes under different time steps.

**Fig 12:**
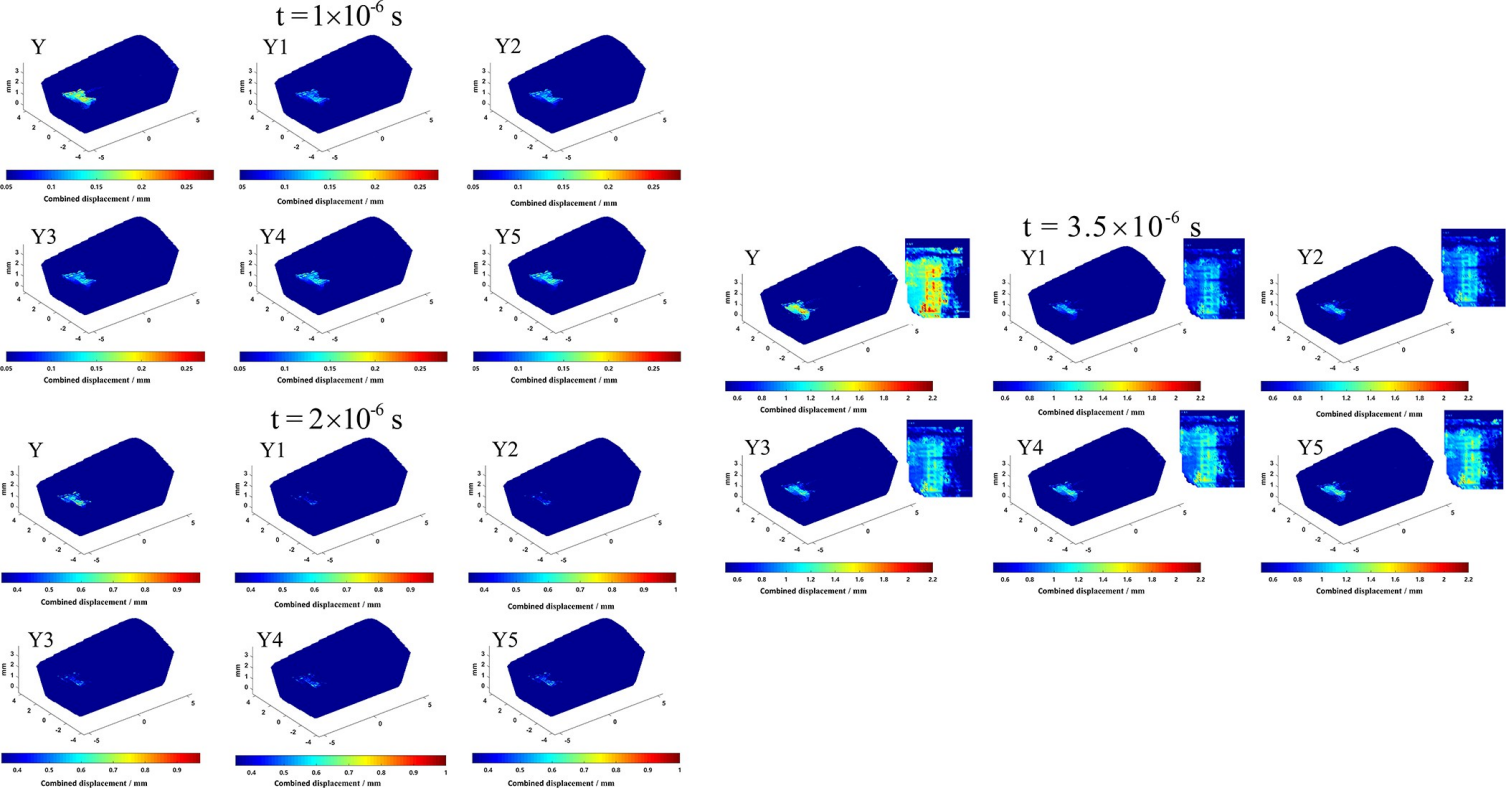
Tool combined displacement changes under different time steps.

Crack visualization is an important analytical method, which can observe and analyze the crack morphology and structure in materials. However, due to the small crack size, complex generation, and evolution process, various shapes and scales, and various types of characterization, crack visualization faces challenges in terms of difficult observation and analysis of diverse crack shapes, data acquisition, and processing accuracy. To overcome the difficulties in crack visualization, this paper uses the MATLAB visualization module to study the maximum direction of force (feed direction) and the magnitude of displacement of material points and uses the magnitude of displacement of material points to characterize the occurrence of cracks. Through four-dimensional visualization processing, a three-dimensional model of cracks is successfully generated. To clearly reflect the difference in the damage before and after the tool optimization, in the process of observing the crack size under different time steps, we set two important conditions: when the change in the position of the material point feed direction exceeds a certain threshold, and its combined displacement exceeds another certain threshold, we can observe the generation of cracks on the tool surface. The use of Y to indicate the non-optimized milling cutter, Y1, Y2, Y3, Y4, and Y5 according to the comprehensive performance of the tool from the best to the worst in turn, the size of the crack generated by different tools is shown in Figs 11 and 12.

As can be seen from Figs 11 and 12, where the direction of feed displacement and combined displacement of the tool are larger (obvious cracks), they are concentrated in the near-field of the main cutting edge, diffused from the main cutting edge to the rake face, and the rake face displacement is widely distributed, which is because the main cutting edge, as the main cutting part of the tool, directly participates in the cutting work and bears greater cutting force and stress. It is relatively easier to crack than other parts, because the energy of cracking is much greater than the energy required for crack propagation, so the diffusion of cracks is easy to form in the near-field of the main cutting edge, thereby reducing the service life of the tool. When the milling time reaches 3.5×10^−6^ s, it can be observed that the cracks of the front cutter face and the main cutting edge are obviously reduced after optimization.

## Analysis and discussion

### Near-field of cutting edge

**Fig 13:**
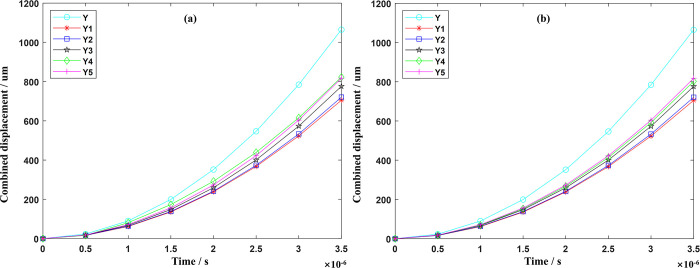
Change combined displacement of critical material points. (a) Tool major cutting edge (b) Tool minor cutting edge.

**Fig 14:**
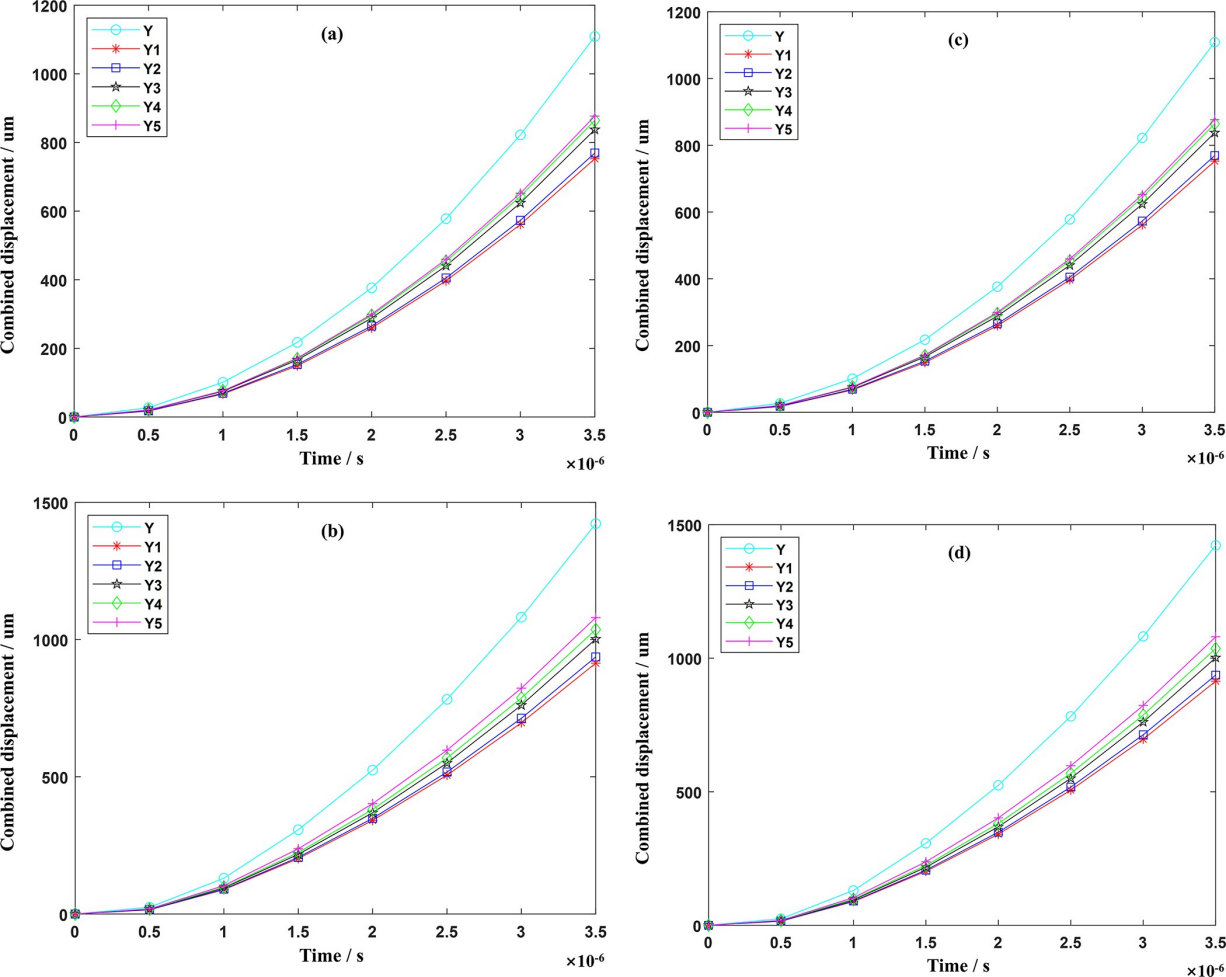
Change combined displacement of rake face and major flank. (a) Rake face (b) Major flank.

**Fig 15:**
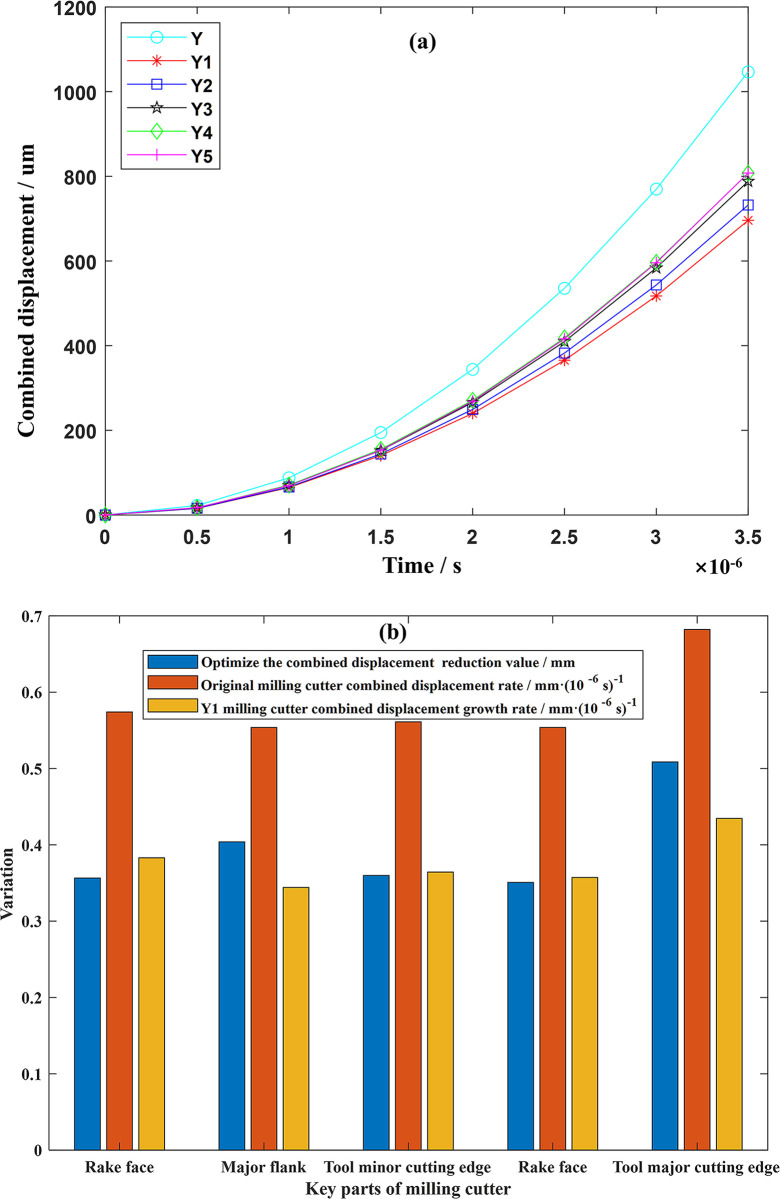
Change in the combined displacement of tool tip and key parts. (a) Tool tip (b) Key parts.

Material points at five key positions of the optimized front and rear tool main cutting edge, tool minor cutting edge, rake face, major flank, and tool tip were tracked respectively, and the results were shown in Figs 13–15.

**Fig 16:**
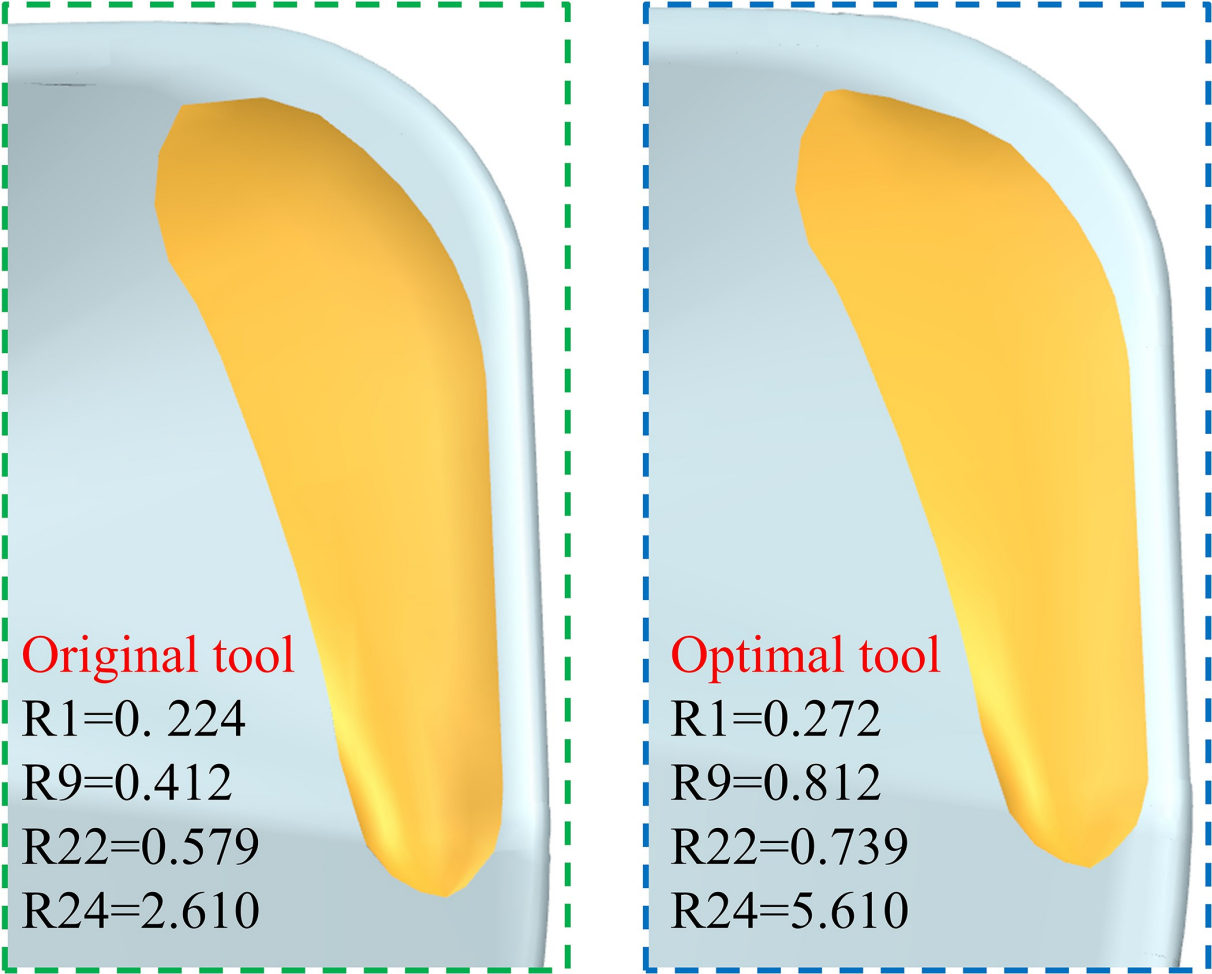
Cutting tool optimization comparison.

During the numerical simulation process, when the time reaches an extremely short 3.5×10^−6^ s, the displacement changes of the material points at the five key positions of the original milling cutter all exceed 1 mm, and the change rate exceeds 0.55 mm/10^−6^ s, which will cause further crack propagation and the tool surface will show obvious crack phenomenon. Therefore, this article regards this time as the time when the impact load is applied and observes the occurrence of tool cracks during this period. The displacement change of the main cutting edge is the most significant, increasing to 1.4228 mm, with a growth rate of 0.682 mm/10^−6^ s. The combined displacement increments of the tool tip prone to impact is 1.0467 mm with a growth rate of 0.554 mm/10^−6^ s. After optimization, the combined displacement of the main cutting edge and the tool tip is reduced by 33.75% and 33.51% respectively, and crack expansion is effectively suppressed. The optimized milling cutter is shown in Fig 16.

**Fig 17:**
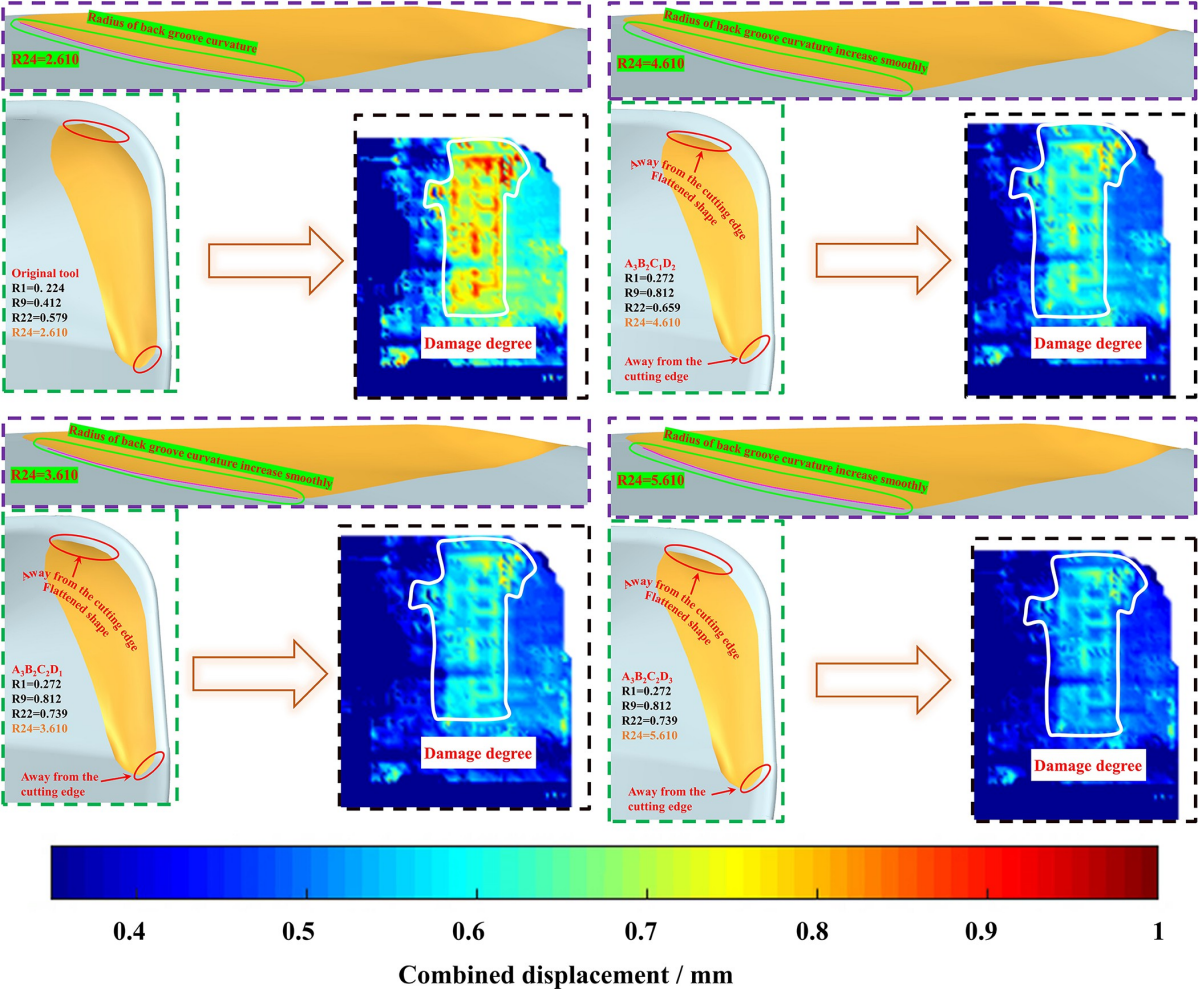
Change of microstructure and damage degree of cutting tool.

Based on PD numerical simulations and simulations, it is observed that within an appropriate range, increasing the inner radius of curvature results in a greater distance between the groove and the cutting edge, causing the groove to project onto the tool surface in a flattened manner. Simultaneously, facilitating a smooth transition between the top and bottom of the groove located at the cutting edge helps reduce evaluation indicators, decrease combined displacement near the cutting edge, and improve the tool’s resistance to damage. The correlation model between the tool microstructure and the degree of damage (combined displacement threshold) is shown in Fig 17.

### Selection of optimal tool scheme

**Fig 18:**
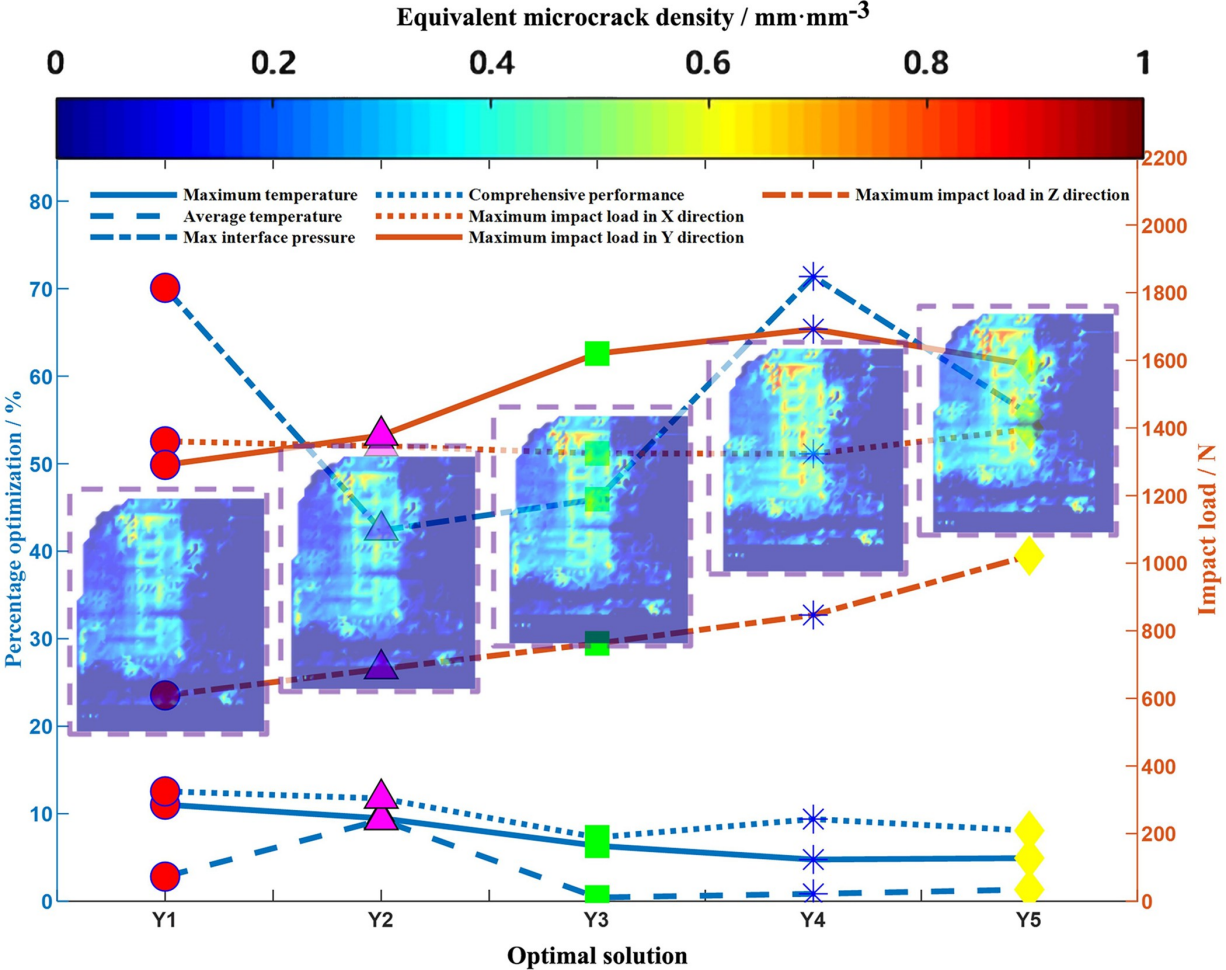
Optimal solution performance optimization—mechanical load—PD damage mapping.

To explore the relationship between impact load and PD damage and verify whether the advantages and weaknesses of the selected scheme are consistent with the simulation, a complete Deform simulation is carried out on the selected five tool optimization schemes under the same working conditions. In PD numerical simulation, when the tool is working for 3.5×10^−6^ s under the maximum impact load, it is set that when the combined displacement of the material points exceeds 0.6 mm, micro-cracks can be observed on the tool surface, and the equivalent micro-crack density is calculated by the simplified formula (equivalent microcrack density = equivalent coefficient × (combined displacement magnitude—threshold)), where the equivalent coefficient is taken as 0.8. Therefore, the mapping model of cutting edge near-field microstructure optimization scheme—mechanical load—damage degree—tool comprehensive cutting performance is obtained, and the results are shown in Fig 18. This model can further strengthen the understanding and judgment of the comprehensive performance of the tool. The degree of tool damage in Fig 18 is consistent with the pros and cons of the scheme. However, it is worth noting that in the experiment, there are often different evaluation indicators, subjective judgment of the feasibility of the experiment and whether the experimental needs are met, and other factors, while ignoring the impact of the interaction. These factors may lead to errors, which have a sudden impact on optimal scheme Y4, making the comprehensive performance of optimal scheme Y4 better than optimal scheme Y3. Nevertheless, the evaluation method is consistent with the overall merits and demerits of the experiment, so the evaluation method has high accuracy and feasibility.

## Conclusion

In this paper, the NX 3D modeling software is used to develop a general discrete body plug-in for discrete geometric models with complex surfaces, and the micro-slot structure of the milling cutter is optimized, the optimal tool scheme is obtained, and the micro-slot control mechanism is analyzed. A milling simulation model based on peridynamics theory was established, and a PD numerical simulation of H13 steel under impact load during milling was successfully conducted. The evolution law of cutting tool cracks during the milling of H13 steel is revealed through comparative analysis of milling cutters before and after optimization. The main conclusions are as follows:

1. This paper optimizes the structure of micro-grooves on the rake face, greatly improving the comprehensive milling performance of the tool, and analyzes the control mechanism of the micro-grooves. Simulation results show that the evaluation index can be effectively reduced by appropriately increasing the distance between the groove on the tool’s outer contour and the cutting edge and projecting it on the tool surface in a flat shape. At the same time, by making the groove depth change gently at the minor cutting edge and increasing the groove depth at the main cutting edge.
2. Based on PD theory, the crack evolution of the tool under impact load during milling was numerically simulated. By comparing the crack propagation of milling cutters before and after optimization, it was found that in five key positions (tool main cutting edge, tool minor cutting edge, rake face, major flank, and tool tip), cracks are prone to form and spread between the near-field of the main cutting edge and the rake face. When the milling time reached 3.5×10^−6^ s, the combined displacement growth at the main cutting edge was the most rapid, reaching 1.4228 mm with a growth rate of about 0.68 mm/10^−6^ s. The optimization effect was most significant at the major flank, with a reduction in combined displacement of about 37.06%.

1. The screening of the superiority and inferiority of the PD analysis results, and the orthogonal simulation experimental scheme resulted in a phenomenon where the comprehensive performance improvement of the Y4 tool was greater than that of the Y3 tool due to subjective judgment of the experimental feasibility and the neglect of factors such as the impact of interaction. Nevertheless, the overall superiority and inferiority of this evaluation method are consistent with those of PD numerical analysis experiments, so this evaluation method has high accuracy and feasibility.

## Supporting information

S1 Data Simulation scheme and results. (XLSX)

S2 Data Key points change rate and reduction value for combined displacement. (XLSX)

S3 Data Key points combined displacement. (XLSX)

S4 Data Optimal solution performance optimization—mechanical load. (XLSX)
